# Treatment of Rickets

**Published:** 1844-04

**Authors:** A. W. Close

**Affiliations:** Manchester


*Treatment of Rickets.*—By A. W. Close, Esq., Manchester. This gentleman states that the softened state of the bones in this affection is owing originally to a deficiency in the supply of *the nutritive nitrogenized substances.* The affection is seldom seen during suckling, because the milk contains those elements which are exactly suited to the wants of the system. After weaning, the diet often adopted among the poor, consists chiefly of potatoes, oatmeal, gruel, tea, coffee, and rice. Now *proteine* is only found in the two first in small quantities, and none in the rest. Among the middle and upper classes the diet after weaning is often sago, rice, or arrowroot, which certainly fatten little children, but do not convey a sufficient quantity of nitrogen to the system. The diet ought to consist more of the nitrogenized substances when there is this disposition in the system, such as beef-tea, eggs, and wheat ground and made into bread without separation of the cuticle of the grain, in which is contained the phosphate of lime, to whose absence the softened condition of the bones is usually attributed.—*Med. Times, Aug.* 19, 1843, *p.* 335. *Braithwaite's Med. Retrospect, No.* 8, *p.* 78.

We have under treatment, at this time, a case illustrative of the truth of the above remarks, upon the errors in the diet of children. In this instance, Rachitis was not the result, but a condition of the system equally deplorable. The patient, a child three years old, since the time of weaning, has lived upon a diet of *sugar*, to the almost total exclusion of nitrogenized substances. The result has been, protracted scorbutus, with all its attendant evils. The little sufferer is still fat and round, though from frequent hemorrhages and suffering, reduced to a state of complete anemia.—Ed.

